# Journey to complete remission of dysplasia and intestinal metaplasia after ESD and EMR of Barrett’s esophagus-related neoplasia

**DOI:** 10.1055/a-2422-2815

**Published:** 2025-05-12

**Authors:** Abel Joseph, Kornpong Vantanasiri, Rohit Goyal, Nikita Garg, Cadman Leggett, D. Chamil Codipilly, Kenneth Wang, William S. Harmsen, John J. Vargo, Sunguk Jang, Prasad Iyer, Amit Bhatt

**Affiliations:** 1 2569 Internal Medicine, Cleveland Clinic Foundation, Cleveland, United States; 2 6429 Division of Gastroenterology and Hepatology, Stanford University, Stanford, United States; 3 6915 Barrett's Esophagus Unit, Division of Gastroenterology and Hepatology, Mayo Clinic, Rochester, United States; 4 Biostatistics, Mayo Clinic, Rochester, MN, USA, Rochester, United States; 5 Section of Therapeutic Endoscopy, Department of Gastroenterology and Hepatology, Digestive Disease Surgical Institute, Cleveland Clinic Foundation, Cleveland, United States; 6 2569 Section of Therapeutic Endoscopy, Department of Gastroenterology and Hepatology, Digestive Disease Surgical Institute, Cleveland Clinic, Cleveland, United States

**Keywords:** Endoscopy Upper GI Tract, Barrett's and adenocarcinoma, Precancerous conditions & cancerous lesions (displasia and cancer) stomach, Endoscopic resection (ESD, EMRc, ...)

## Abstract

**Background and study aims:**

Although endoscopic submucosal dissection (ESD) is associated with higher en-bloc and R0 resection rates than cap-assisted endoscopic mucosal resection (cEMR), its comparative impact on achieving complete remission of dysplasia (CRD) and intestinal metaplasia (CRIM) in BE endoscopic eradication therapy (EET) is not well defined. We aimed to compare the journey of patients from initial endoscopic resection (ER) with ESD and cEMR to achieving CRD and CRIM.

**Patients and methods:**

Patients undergoing ESD or cEMR followed by ablation for BE neoplasia at two academic institutions in the United States were included. Primary outcomes included CRD and CRIM rates following ER in the two groups. Secondary outcomes included the number of resection/ablative procedures from initial ER to achieving CRD and CRIM. Inverse probability treatment weighting (IPTW) was used to balance confounding variables between groups.

**Results:**

A total of 801 patients (606 cEMR, 195 ESD) were included. ESD group patients had higher en-bloc resection rates (ESD 94.4%, cEMR 44.7%). Higher rates of CRD were observed in patients undergoing initial ESD (HR 1.53, *P* < 0.01). With time-to-event and IPTW analyses, rates of achieving CRD and CRIM were comparable between the groups. There were no significant differences in mean number of endoscopic resection or ablative procedures among patients undergoing initial cEMR resection compared with those treated with initial ESD.

**Conclusions:**

Despite larger lesion sizes and more cancers in patients undergoing ESD, the EET journey to achieving CRD and CRIM was comparable to that in patients receiving cEMR. Prospective studies are required to further study differences between these two treatment approaches.

## Introduction

In treatment of many gastrointestinal epithelial tumors, endoscopic submucosal dissection (ESD) achieves resection with higher en-bloc and R0 resection rates than cap-assisted endoscopic mucosal resection (cEMR) 1 . This has been demonstrated in gastric and esophageal squamous cell cancers. In treatment of colonic lateral spreading tumors, ESD has also been shown to achieve higher R0 resection and lower recurrence rates than piecemeal EMR 2 . Early Barrett’s esophagus (BE) dysplasia/neoplasia differs from the tumors mentioned above, in that it develops in a field of precancerous changes of surrounding metaplasia. Endoscopic eradication therapy (EET) has become standard of care for patients with BE and dysplasia to prevent development of esophageal adenocarcinoma 3 4 . The two main techniques used for endoscopic resection (ER) in EET of BE dysplasia and neoplasia are cEMR and ESD.

In treatment of early BE neoplasia, ESD has been shown to achieve higher en-bloc and R0 resection rates than piecemeal EMR 5 6 . However, endoscopic treatment of early BE neoplasia involves not only resection of focal lesion, but also ablation of the remaining BE mucosa until complete remission of dysplasia (CRD) and complete remission of intestinal metaplasia (CRIM) are achieved 7 8 . Studies have shown that if CRIM is not achieved, there are significantly higher recurrence rates 9 . Prior studies have shown somewhat higher complication rates with ESD compared with cEMR in BE case series 5 10 11 , with high rates of achieving CRIM with both cEMR and ESD followed by ablation 12 . However comparative data between these two approaches are scarce 12 . This is important given the challenges with wider dissemination of ESD training and practice in the West.

The question of whether the benefit of higher en-bloc and R0 resection rates with ESD translates to clinical outcomes such as CRIM in the goal of EET remains unanswered. Hence, we aimed to compare the journey of patients with early BE neoplasia undergoing initial ER with cEMR or ESD followed by endoscopic ablation to achieve CRD and CRIM in a large multicenter North American cohort. Our primary outcomes were rates and time to CRD/CRIM following initial ER. Our secondary outcomes were number of endoscopic procedures from initial cEMR or ESD to achieving CRD and CRIM.

## Patients and methods

The study protocol was approved by the Institutional Review Board of each participating center and the requirement for informed consent was waived because of the retrospective nature of the study.

### Patient selection and data collection

The study was a retrospective analysis of a two-center North American cohort of patients who underwent EET for management of BE or esophageal adenocarcinoma (EAC) from January 2006 to November 2022. Patients were included if they underwent either cEMR or ESD, followed by endoscopic ablation for management of dysplastic BE or EAC. Patients who underwent both cEMR and ESD at initial resection procedure or received surgery or chemoradiation before or after endoscopic therapy were excluded from the analysis. Differences in background flat BE mucosa before EET between the two groups were not studied. Clinical information studied included demographics, size of visible lesions, treatment details, histology, and date of last follow-up evaluation.

### Methods of endoscopic eradication therapy

More than 98% of resection procedures during the study period were performed by five endoscopists (P.G.I., K.K.W., J.V., S.J. and A.B.) with considerable expertise in EET for BE neoplasia. Patients received general anesthesia, sedation with propofol, or conscious sedation. Patients typically were discharged after the procedure, although in certain instances (after complex resections or in very elderly patients), patients were admitted overnight for observation and discharged the next morning.

Standard diagnostic or therapeutic endoscopes were used (Olympus, Center Valley, Pennsylvania, United States; or Fujinon-Fujifilm Medical Systems USA, Lexington, Massachusetts, United States) for cEMR. Standard diagnostic (Olympus, Center Valley, Pennsylvania, United States; or Fujinon-Fujifilm Medical Systems USA, Lexington, Massachusetts, United States) or the Olympus water jet endoscope (180J) were used for ESD. Lesions were assessed carefully with narrow-band imaging (including the near-focus mode) and marked circumferentially with cautery before resection. In general, cEMR was used for lesions less than 1.5 to 2 cm in diameter and ESD was used for larger lesions. ESD has been used at both institutions since 2015. Choice of initial ER technique (cEMR or ESD) was at the discretion of the treating endoscopist. Choice of ablation technique (radiofrequency ablation, cryotherapy, and argon plasma coagulation) after complete resection of visible lesions was also at the discretion of the treating endoscopist.

### Cap-assisted endoscopic mucosal resection

We previously described the cEMR technique 13 . A hard cap (EMR001; Olympus USA) was fitted on the end of the endoscope. A saline and epinephrine solution was injected submucosally under the lesion of interest. A crescent-shaped snare (SnareMaster Crescent; Olympus USA) was seated on the inner aspect of the cap, followed by suction of the lesion into the cap, closure of the snare, and resection using a combination of cutting and coagulation current from an electrosurgical generator (Conmed Beamer; Conmed USA, Utica, New York, United States or VIO 300D, ENDO CUT Q; Erbe USA, Inc, Marietta, Georgia, United States). For band-ligator EMR, either the Duette Multi-Band Mucosectomy System (Cook Medical, Bloomington, Indiana, United States) or the Captivator EMR System (Boston Scientific, Marlborough, Massachusetts, United States) was used. In both kits, a preloaded band ligator was attached to the endoscope to allow for sequential banding of mucosa. Afterward, a hexagonal snare was used to resect the banded tissue with electrocautery.

### Endoscopic submucosal dissection

Standard diagnostic endoscopes (Olympus, Center Valley, Pennsylvania, United States; or Fujinon-Fujifilm Medical Systems USA, Lexington, Massachusetts, United States) or the Olympus water jet endoscope (180J) were used with a soft plastic cap attached to the end of the endoscope. A submucosal injection fluid was injected beneath the lesion. Choice of submucosal injection fluid was at the discretion of the endoscopist and included hydroxyl methyl propyl cellulose solutions, Orise gel (Boston Scientific, USA), Eleview (Medtronic, USA), or Hydroxyethyl starch. Incision and dissection were performed with endoscopic knives, including the DualKnife (Olympus USA), HookKnife (Olympus USA), IT Nano Knife (Olympus USA), Orise ProKnife (Boston Scientific USA) or Clutch Cutter (Fujifilm Medical USA) at endoscopist discretion 14 . A standard electrosurgical generator (VIO 300D, ENDO CUT Q; Erbe USA, Inc, Marietta, Georgia, United States) was used with appropriate current settings as previously described 15 16 .

### Histopathological analysis

EMR specimens were submitted for histopathology in formalin jars. ESD specimens were pinned to a Styrofoam or cork board for formalin fixation and histopathologic examination. All ER specimens underwent standard histopathology processing, analysis, and interpretation by at least one highly experienced, expert gastrointestinal pathologist at both institutions. All specimens were sectioned serially using a bread loaf sectioning technique and underwent standard hematoxylin and eosin staining. Slides were analyzed for lesion size, histology, and involvement of resection margins (lateral and deep). En-bloc resection was defined as a resected specimen submitted to pathology in one piece. R0 resection was designated to the resected ESD specimens with histologic absence of the highest histologic grade within the lesion (EAC/high-grade dysplasia [HGD]/low-grade dysplasia [LGD]) at both deep and lateral margins.

### Follow-up evaluation

After initial ER, patients underwent endoscopic surveillance with occasional biopsies at 3-month intervals until CRD and CRIM were achieved. Careful inspection of the esophageal mucosa was performed with both high-definition white-light endoscopy (WLE) and narrow-band imaging (NBI). Surveillance biopsy specimens of visible mucosal abnormalities were obtained. The majority of patients had biopsies taken to confirm CRIM/CRD, while in others, the diagnosis was made on visual inspection. Biopsies were not taken if visual inspection (with NBI and high-definition WLE) did not show residual BE. Ablation was accomplished using radiofrequency ablation (RFA) (BarrX device; Medtronic, Minneapolis, Minnesota, United States), liquid nitrogen cryoablation (TruFreeze spray cryotherapy system; Steris, Mentor, Ohio, United States), or balloon cryoablation (CryoBalloon Focal Ablation System; Pentax Medical, Montvale, New Jersey, United States). This was applied using standard manufacturer-recommended methodology.

### Primary and secondary outcomes

Primary outcomes were rate and time to CRD (defined as absence of dysplasia on biopsies from the tubular esophagus and gastroesophageal junction during at least one surveillance endoscopy after the initial resection and ablative procedures) and rate and time to CRIM (defined as absence of intestinal metaplasia on biopsies and visible BE from the tubular esophagus and gastroesophageal junction during at least one surveillance endoscopy after the initial resection and ablative procedures). Our secondary outcome was the number of endoscopic procedures including both ER and ablative procedures from initial cEMR or ESD to achieving CRD and CRIM.

### Statistical analysis

Statistical analysis was performed using SAS version 9.4. Descriptive statistics were used to summarize patient demographics, endoscopic findings, pathology, procedure details, and follow-up outcomes. Differences between groups were analyzed using Fisher's exact test or chi-squared test for categorical variables and Student's *t* -test or Wilcoxon rank-sum test for continuous variables. Kaplan-Meier analysis was used to estimate time to achieving CRD and CRIM.

Median (interquartile range [IQR]) follow-up to CRIM and CRD have been reported using the reverse Kaplan-Meier method. Where no estimate was available, we have reported as “NR,” not reached at time of the analyses of the cohort.

To decrease risk of confounding in the setting of non-randomized nature of this comparative study, inverse probability of treatment weighting (IPTW) using propensity scores was used to compare the two treatments, cEMR vs ESD. IPTW analysis is a statistical method used to mimic randomized situations in which both treatment groups are allocated in balance by creating a pseudo-population in which the measured and known confounders are equally distributed across both treatment groups. To estimate the propensity score, logistic regression analysis was performed using the treatment group (cEMR vs ESD) as the dependent variable and age, sex, hiatal hernia length, maximal BE length, baseline worst-case histology, and lesion size as independent variables. The IPTW method based on propensity scoring was then used to estimate the average treatment effect of cEMR compared with ESD. A Cox proportional hazards model was used to identify associations of treatment with time to achieving CRD and CRIM accounting for IPTW weights using a robust sandwich estimator. The association of treatment with outcomes was also examined using a multivariable Cox model including the clinically relevant covariates of age, sex, body mass index, hiatal hernia length, maximal BE length, baseline worst-case histology (HGD or EAC versus otherwise), maximal length of BE per Prague classification, and lesion size. Because there were 597 CRIM events and 723 CRD events, all clinical variables collected possibly associated were included as covariates in the multivariable models. A two-sided *P* < 0.05 was considered statistically significant.

## Results

**Fig. 1:**
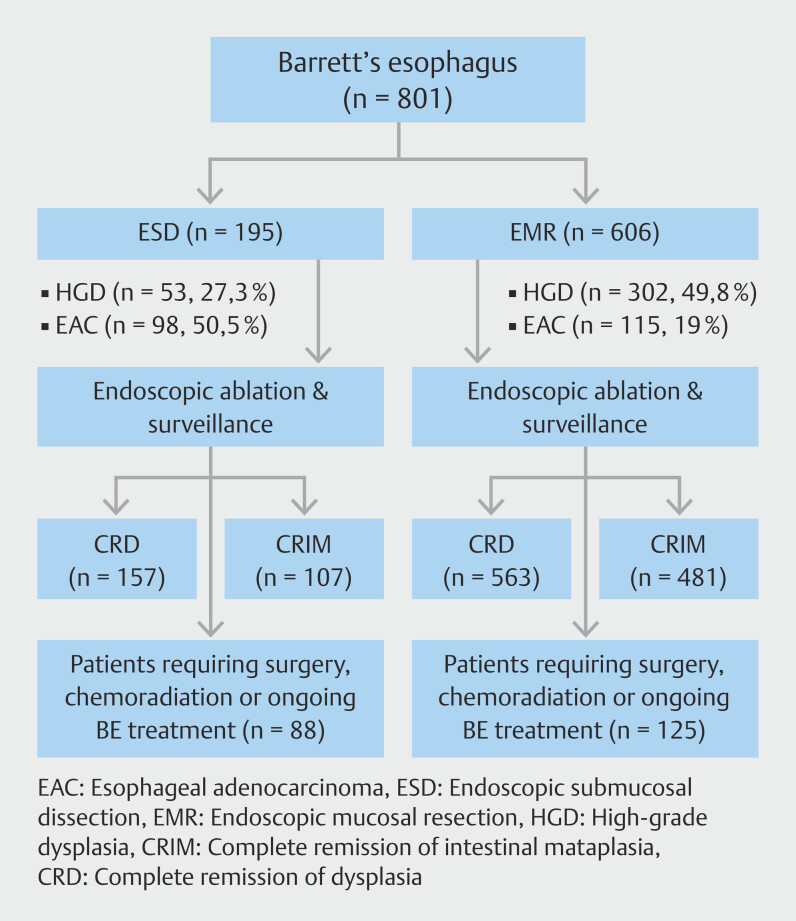
Flowchart diagram of the study.

Patient flow in the study is shown in Fig. 1 . From January 2006 to November 2022, we identified 801 patients who underwent either cEMR or ESD followed by ablation at the two academic institutions. A total of 606 patients underwent cEMR, whereas 195 underwent ESD.

Baseline characteristics of patients in the cEMR and ESD groups are presented in Table 1 . Mean age of patients in the ESD group was higher than in the cEMR group. Mean visible lesion size was also larger in the ESD group. Most of the patients in the cEMR group had HGD (49.8%) followed by EAC (19.0%), whereas most patients in the ESD group had EAC (50.5%) followed by HGD (27.3%). In terms of EAC staging, many patients in both groups were T1a, with no significant difference in T stage between the two groups. En-bloc resection was significantly more common in the ESD group compared with the cEMR group (94.4% vs 44.4%, *P* < 0.01). Patients requiring surgery or chemoradiation were excluded from CRD and CRIM analysis (ESD, n = 88, EMR, n = 125).

**Table:** **Table 1** Baseline group characteristics.

	**Initial cEMR (n = 606)**	**Initial ESD (n = 195)**	***P* value **
Age at procedure, mean (SD)	65.4 (10.0)	69.2 (10.0)	< 0.01*
Sex, males, n (%)	493 (81.4%)	158 (81.0%)	0.91 ^†^
Ethnicity, not Hispanic, n (%)	587 (98.3%)	186 (95.4%)	** < 0.01 ^†^**
BMI, kg/m ^2^ , mean (SD)	30.7 (5.5)	30.0 (5.4)	0.09*
Hiatal hernia, cm	3.1 (2.1)	2.4 (1.9)	<0.01*
Active smoker, n (%)	419 (70.4%)	128 (67.0%)	0.4 ^†^
Lesion size, mm	12.8 (7.1)	27.0 (11.4)	< 0.01*
Maximal length of Barrett’s, cm	5.4 (3.9)	4.9 (3.6)	
Pre-resection histology, n (%)			** < 0.01 ^†^**
No BE	5 (0.9%)	1 (0.5%)	
NDBE	8 (1.4%)	4 (2.1%)	
LGD	90 (15.3%)	9 (4.8%)	
HGD	357 (60.7%)	81 (43.1%)	
EAC	128 (21.8%)	93 (49.5%)	
Post-resection histology of resected specimen, n (%)			** < 0.01 ^†^**
NDBE	62 (10.2%)	8 (4.1%)	
LGD	120 (19.8%)	29 (14.9%)	
HGD	302 (49.8%)	53 (27.3%)	
EAC	115 (19.0%)	98 (50.5%)	
T1a	63 (77.8%)	84 (84.0%)	
T1b	18 (22.2%)	16 (16.0%)	
En-bloc resection, n (%)	271 (44.7%)	184 (94.4%)	** < 0.01 ^†^**
Total follow-up time, months	66.9 (63.13, 70.82)	22.05 (19.94, 24.16)	
*Equal variance t-test. †Chi-square. BE, Barrett’s esophagus; BMI, body mass index; cEMR, cap-assisted endoscopic mucosal resection; EAC, esophageal adenocarcinoma; ESD, endoscopic submucosal dissection; HGD, high-grade dysplasia; LGD, low-grade dysplasia; NDBE, non-dysplastic Barrett’s esophagus; SD, standard deviation.			

### Journey to CRD with endoscopic eradication therapy

**Fig. 2:**
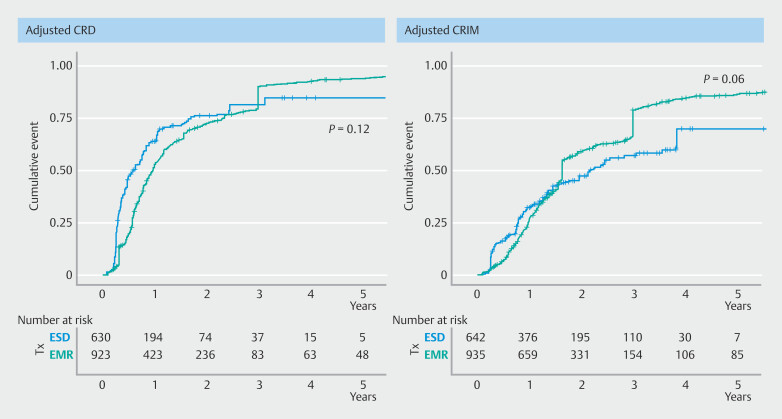
IPTW-adjusted Kaplan-Meier curves for CRD and CRIM outcomes following initial ER.

Overall cumulative probabilities of achieving CRD at 5 years following initial cEMR and ESD were 90.43% (87.59%-92.62%) and 94.93% (89.38%-98.77%), respectively. The IPTW-adjusted Kaplan-Meier curve ( Fig. 2 ) demonstrated that the cumulative probability of CRD in both groups was comparable.

Table 2 presents the journey to CRD after ER therapy. Mean number of ablative procedures was comparable between the groups, with the initial cEMR group undergoing a mean of 1.10 (standard deviation [SD] = 0.65) and the initial ESD group undergoing a mean of 1.07 procedures (SD = 0.7) until CRD. Similarly, regarding the mean number of subsequent ER, the initial ESD group had a slightly higher value (0.41, SD = 0.22) compared with the initial cEMR group (0.35, SD = 0.27). No difference was noted on comparing specific ablative techniques.

**Table:** **Table 2** Journey to CRD with EET between the cEMR and ESD groups.

	**Initial cEMR (n = 555)**	**Initial ESD (n = 168)**	***P* value **
Mean number of endoscopic resections (SD)	0.35 (0.27)	0.41 (0.22)	0.05
Mean number of ablative procedures (SD)	1.10 (0.65)	1.07 (0.7)	0.66
Mean number of endoscopies to CRD	2.1 (2.0)	2.1 (1.6)	0.58
Mean number of all endoscopic procedures to CRD	3.7 (3.9)	3.4 (3.1)	0.35
APC, argon plasma coagulation; cEMR, cap-assisted endoscopic mucosal resection; CRD, complete remission of dysplasia, CRIM, Complete remission of intestinal metaplasia; EET, endoscopic eradication therapy; EMR, endoscopic mucosal resection; ESD, endoscopic submucosal dissection; RFA, radiofrequency ablation; SD, standard deviation.			

### Journey to CRIM after endoscopic eradication therapy

Overall cumulative probabilities of achieving CRIM at 5 years following initial cEMR and ESD were 80.45% (76.62%-83.65%) and 81.68% (69.43%-91.62%), respectively. Predictors of CRD using a multivariable Cox model are presented in Table 3 . Longer maximal length of BE (HR 0.91, *P* < 0.01) and larger hiatal hernia (HR 0.94, *P* = 0.0037) were associated with lower CRD rates. Initial treatment with ESD was associated with higher odds of CRD compared with cEMR (HR 1.53, *P* < 0.01). Table 4 presents results of an IPTW-matched multivariable Cox model examining factors associated with CRD. Longer maximal segment of BE (HR 0.90, *P* < 0.01) and larger hiatal hernia (HR 0.91, *P* < 0.01) were associated lower CRD rates. ( Table 3 , Table 5 ). However, on IPTW analysis, initial ER technique (ESD vs cEMR) was not associated with increased rates of CRD (HR 1.34, *P* = 0.12).

**Table:** **Table 3** Multivariable Cox model without IPTW adjustment identifying predictor of CRD.

		**Hazard ratio (95% CI)**	***P* value **
Sex	Male	1.02 (0.83–1.26)	0.82
	Female	1.0 (reference)	
Age, per 10 years		1.0 (0.93–1.09)	0.90
Prague (M), per 1cm		0.91(0.89–0.93)	**< 0.01**
Worst post-resection histology	No BE/NDBE/LGD	1.08 (0.90–1.28)	0.40
	HGD/EAC	1.0 (reference)	
Hiatal hernia, per 1 cm		0.94 (0.90–0.98)	**< 0.01**
Lesion size, per 1 mm		1.01 (1.01–1.03)	**< 0.01**
BMI, per 1 unit		1.01(0.99–1.03)	0.06
Smoking history	Current/former	1.05 (0.88–1.25)	0.56
	Never	1.0 (reference)	
Procedure type	ESD	1.53 (1.21–1.93)	**< 0.01**
	EMR	1.0 (reference)	
BE, Barrett’s esophagus; CI, confidence interval; CRD, complete remission of dysplasia; CRIM, complete remission of intestinal metaplasia; EAC, esophageal adenocarcinoma; EMR, endoscopic mucosal resection, ESD, endoscopic submucosal dissection; HGD, high-grade dysplasia; IPTW, inverse probability of treatment weighting; LGD, low-grade dysplasia; NDBE, non-dysplastic Barrett’s esophagus.			

**Table:** **Table 4** Multivariable Cox model with IPTW adjustment for CRD identifying predictor of CRD.

		**Hazard ratio (95% CI)**	***P* value **
Sex	Male	0.91 (0.50–1.67)	0.77
	Female	1.0 (reference)	
Age, per 10 years		0.88 (0.77–1.0)	0.06
Prague (M), per 1cm		0.90 (0.85 – 0.95)	**< 0.01**
Worst post-resection histology	No BE/NDBE/LGD	1.2 (0.86 – 1.65)	0.27
	HGD/EAC	1.0 (reference)	
Hiatal hernia, per 1 cm		0.91 (0.85–0.98)	**0.01**
Lesion size, per 1 mm		1.01 (0.99–1.03)	0.07
BMI, per 1 unit		1.00 (0.97–1.03)	0.64
Smoking history	Current/former	0.83 (0.54 – 1.27)	0.38
	Never	1.0 (reference)	
Procedure type	ESD	1.34 (0.92–1.96)	0.12
	EMR	1.0 (reference)	
BE, Barrett’s esophagus; BMI, body mass index; CI, confidence interval; CRD, complete remission of dysplasia; CRIM, complete remission of intestinal metaplasia; EAC, esophageal adenocarcinoma; EMR, endoscopic mucosal resection; ESD, endoscopic submucosal dissection; HGD, high-grade dysplasia; IPTW, inverse probability of treatment weighting; LGD, low-grade dysplasia; NDBE, non-dysplastic Barrett’s esophagus.			

**Table:** **Table 5** Journey to CRIM with EET between the cEMR and ESD groups.

	**Initial EMR (n = 480)**	**Initial ESD (n = 117)**	***P* value **
Mean number of endoscopic resections (SD)	1.56 (1.06)	1.17 (0.60)	**< 0.01**
Mean number of ablative procedures (SD)	1.80 (1.7)	1.65 (1.44)	0.10
Mean number of endoscopies to CRIM	3.3 (2.8)	3.2 (2.5)	0.86
Mean number of any endoscopic procedure to CRIM	5.4 (5.0)	5.0 (4.3)	0.45
cEMR, cap-assisted endoscopic mucosal resection; CRIM, complete remission of intestinal metaplasia; EET, endoscopic eradication therapy; EMR, endoscopic mucosal resection, ESD, endoscopic submucosal dissection; SD, standard deviation.			

Median follow-up time was 66.9 months (IQR, 63.13, 70.82) in the cEMR group and 22.05 months (IQR, 19.94, 24.16) in the ESD group. The IPTW-adjusted Kaplan-Meier curve ( **Fig. 2** ) demonstrated that cumulative probability of CRIM in both groups was comparable.

Predictors of CRIM using a multivariable Cox model are presented in Table 6 . Longer maximal length of BE (HR 0.86, *P* < 0.01) and larger size of hiatal hernia (HR 0.91, *P* < 0.01) were associated with lower CRIM rates. ESD was not associated with higher odds of CRIM compared with cEMR (HR 0.80, *P* = 0.10). Table 7 presents results of an IPTW-matched multivariable Cox model examining factors associated with CRIM. Longer maximal segment of BE (HR 0.84, *P* < 0.01) and larger hiatal hernia (HR 0.84, *P* < 0.01) were associated lower CRIM rates. Larger lesion size was associated with a statistically higher rate of CRIM (HR 1.01, *P* = 0.01). On IPTW analysis, initial ESD was associated with a slightly lower probability of CRIM compared with cEMR with borderline statistical significance (ESD vs cEMR: HR 0.71, *P* = 0.06).

**Table:** **Table 6** Multivariable Cox model without IPTW adjustment identifying predictors of CRIM.

		**Hazard ratio (95% CI)**	***P* value **
Sex	Male	1.36 (1.07–1.72)	0.01
	Female	1.0 (reference)	
Age, per 10 years		0.98 (0.89–1.08)	0.75
Prague (M), per 1cm		0.86 (0.84–0.89)	**< 0.01**
Worst post-resection histology	No BE/NDBE/LGD	0.89 (0.74–1.08)	0.26
	HGD/EAC	1.0 (reference)	
Hiatal hernia, per 1 cm		0.91 (0.87 – 0.95)	**< 0.01**
Lesion Size, per 1 mm		1.02 (1.01–1.03)	**< 0.01**
BMI, per 1 unit		1.01 (0.99–1.02)	0.12
Smoking history	Current/Former	0.93 (0.77–1.12)	0.46
	Never	1.0 (reference)	
Procedure type	ESD	0.80 (0.61–1.07)	0.10
	EMR	1.0 (reference)	
BE, Barrett’s esophagus; BMI, body mass index; CI, confidence interval; CRD, complete remission of dysplasia; CRIM, complete remission of intestinal metaplasia; EAC, esophageal adenocarcinoma; EMR, endoscopic mucosal resection; ESD, endoscopic submucosal dissection; HGD, high-grade dysplasia; IPTW, inverse probability of treatment weighting; LGD, low-grade dysplasia; NDBE, non-dysplastic Barrett’s esophagus.			

**Table:** **Table 7** Multivariable Cox model with IPTW adjustment identifying predictors of CRIM.

		**Hazard ratio (95% CI)**	***P* value **
Sex	Male	1.27 (0.91–1.75)	0.14
	Female	1.0 (reference)	
Age, per 10 years		0.96 (0.82–1.12)	0.61
Prague (M), per 1cm		0.84 (0.81–0.88)	**< 0.01**
Worst post-resection histology	No BE/NDBE/LGD	0.86 (0.61–1.19)	0.36
	HGD/EAC	1.0 (reference)	
Hiatal hernia, per 1 cm		0.84 (0.79–0.90)	**< 0.01**
Lesion size, per 1 mm		1.01 (1.00–1.02)	**< 0.01**
BMI, per 1 unit		1.00 (0.98–1.03)	0.51
Smoking history	Current/former	0.67 (0.5–0.90)	**< 0.01**
	Never	1.0 (reference)	
Procedure type	ESD	0.71 (0.5–1.01)	0.06
	EMR	1.0 (reference)	
BE, Barrett’s esophagus; BMI, body mass index; CI, confidence interval; CRD, complete remission of dysplasia; CRIM, complete remission of intestinal metaplasia; EAC, esophageal adenocarcinoma; EMR, endoscopic mucosal resection; ESD, endoscopic submucosal dissection; HGD, high-grade dysplasia; IPTW, inverse probability of treatment weighting; LGD, low-grade dysplasia; NDBE, non-dysplastic Barrett’s esophagus.			

Table 5 presents results of the journey to CRIM after ER therapy. There was no significant difference in mean number of repeat ERs until CRIM between ESD and cEMR (1.56 vs 1.17). Mean number of ablations to CRD was also similar in the Initial ESD group compared with the Initial cEMR group (1.65 vs 1.80). Mean number of endoscopies to CRIM and mean number of all endoscopic procedures before CRIM were not significantly different between the two groups (3.3 vs 3.2, *P* = 0.86 and 5.2 vs 5.6, *P* = 0.45, respectively). No difference was noted on comparing specific ablative techniques.

### A priori subgroup analysis in patients with high-risk histology

To assess the rate of CRD and CRIM in patients with high-risk histology, an analysis comparing cEMR (N = 418) to ESD (N = 152) in patients who had post-resection worst-case histology of HGD or EAC was also performed. Subgroup analysis demonstrated that odds of reaching CRD were comparable between the two groups (ESD vs cEMR: HR 1.16; 95% confidence interval [CI] 0.65–2.08; *P* = 0.61). Similarly, risk of reaching CRIM were also comparable between the two ER techniques (ESD vs cEMR: HR 0.84; 95% CI 0.54–1.31; *P* = 0.44).

## Discussion

In this large retrospective cohort study from two academic medical centers, we investigated comparative outcomes of EET with cEMR or ESD followed by endoscopic ablation in patients with dysplastic BE and EAC. The journey to achieving CRD and CRIM was largely similar between the two groups. On IPTW analysis, initial ESD was associated with lower probability of CRIM compared with cEMR, with borderline statistical significance. This could have been related to more advanced pathology in the ESD arm. This idea is supported by our subgroup analysis looking at high-risk pathology, where no difference was seen between techniques in achieving CRIM. In addition, there were no significant differences in number of additional ER and ablative procedures to reach CRIM/CRD between the two groups. ESD is a more technically demanding procedure than cEMR and is usually reserved for larger and more complex lesions that may be difficult to remove completely with cEMR alone. At the two centers included in this study, both EMR and ESD procedures are performed, with smaller easier lesions preferentially undergoing cEMR, and larger, bulky, or scarred lesions undergoing ESD. Despite this, similar journeys to CRD and CRIM were seen in both groups.

In a recent single-center retrospective study by Codipilly et al., it was reported that ESD achieved a higher rate of CRD than cEMR (85.6% vs 75.8%) at 2 years despite larger lesion size and more advanced histology. Two retrospective studies also did not show a significant difference in CRIM rate between the two ER techniques 17 18 19 . It is worth noting that all of these studies assessing CRIM rate were retrospective in nature including variable definitions with significantly shorter follow-up time in the ESD group, suggesting a moderate degree of length time bias. We recently found that ESD of more advanced HGD or EAC results in more definitive treatment with lower recurrence rates and less need for repeat endoscopic interventions. However, in this study, recurrence was defined as any visible dysplasia or cancer occurring immediately after initial ER 5 . A small randomized controlled trial (RCT) compared histologic outcomes of cEMR with ESD, followed by ablation in patients with dysplastic BE and early EAC 20 . This study defined CRD as histologic absence of HGD or EAC on at least one surveillance endoscopy after the initial ER procedure, and CRIM rate was not included as the study outcome. Although ESD group achieved a significantly higher rate of en-bloc R0 resection, there was no difference in the rate of CRD at 3 months. Findings from our study further elaborate and substantiate these earlier findings, showing similar CRD rates between the two ER techniques (particularly after adjustment with IPTW).

We hypothesized that ESD should reduce need for additional endoscopic treatment procedures, given the larger resected ESD specimen, leaving less residual BE mucosa to be treated. However, we found that the ESD group required a similar number of additional endoscopic treatments (both ER and ablative procedures) compared with the cEMR group to achieve CRD (3.4 vs 3.7 sessions; *P* = 0.35) and CRIM (5.0 vs 5.4 sessions; *P* = 0.45). This likely reflects more EACs in the ESD group, possibly resulting in higher numbers of supplementary endoscopic treatment sessions to achieve CRD and CRIM. In addition, 38% of patients (8/21) receiving repeat ER after initial ESD had benign pathology such as non-dysplastic BE or BE with LGD on repeat ER after initial ESD, helping explain the higher number of additional ER procedures than expected in the ESD group. This is likely reflective of the paradigm for resecting any subtle visible or nodular tissue in BE neoplasia treatment.

Moreover, it is also possible that lack of statistical difference in this outcome is explained by a type 2 error, with a larger sample size required to detect subtle differences in this particular outcome. However, findings from our study seem to be consistent with what has been reported in previous literature. In a retrospective comparative study by Subramaniam et al 17 , the study suggested that the outcome for management of early BE neoplasia between cEMR and ESD followed by ablation were similar in regard to the number of subsequent ablative procedures after initial ER.

This study has several strengths. First, it included patients from two high-volume academic BE expert centers, with expertise in management of these patients. Second, the study included patients who underwent either cEMR or ESD followed by additional endoscopic therapies, which allowed for direct comparison between the two ER techniques and reflected real-life scenarios in clinical practice. Third, the study included a relatively long follow-up period, which allowed for assessment of long-term outcomes of the different treatment modalities. Finally performing IPTW analysis enabled robust adjustment for confounding variables such as lesion size in addition to multivariable Cox proportional models. This study has some limitations. First, it was retrospective, which can be subject to selection bias, length time, and confounding biases. Another limitation of our study is that patients in the ESD group had larger lesions and more advanced histology than the cEMR group. While we attempted to use IPTW analysis to standardize for these differences among the two groups, there are some inherent differences not captured in these statistical methods, such as adverse events, lesion complexity, and available expertise. For instance, ESD was performed on many patients with visible lesions not amenable to cEMR. ESD was introduced later than cEMR, leading to differences in experience with ER of residual Barrett’s mucosa and improved endoscope quality over the study duration, potentially influencing outcomes for patients included later in the study period.

## Conclusions

Despite these issues, our study showed that BE patients undergoing initial resection with ESD were able to achieve CRD/CRIM at a similar rate and timeframe to cEMR. Findings from our study provide several important insights into the effectiveness of ER for management of BE and EAC. It also underscores the importance of ongoing surveillance and monitoring for recurrence, particularly in patients with more advanced BE neoplasia who may require more intensive treatment. Further evaluation through RCTs between ESD and cEMR in treatment of BE neoplasia are ongoing (clinicaltrials.gov, NCT03427346, NCT05276791).

## Supplementary Material

Supplementary Material Zusatzmaterial
