# Supplementary material for: Journey to complete remission of dysplasia and intestinal metaplasia after ESD and EMR of Barrett’s esophagus-related neoplasia
Source: Endosc Int Open. 2025 May 12;13:a24222815. doi: 10.1055/a-2422-2815 (PMC12080518; doi:10.1055/a-2422-2815)

Supplementary Figure 1 Kaplan-Meier curves for CRD and CRIM outcomes without IPTW adjustment following initial ER

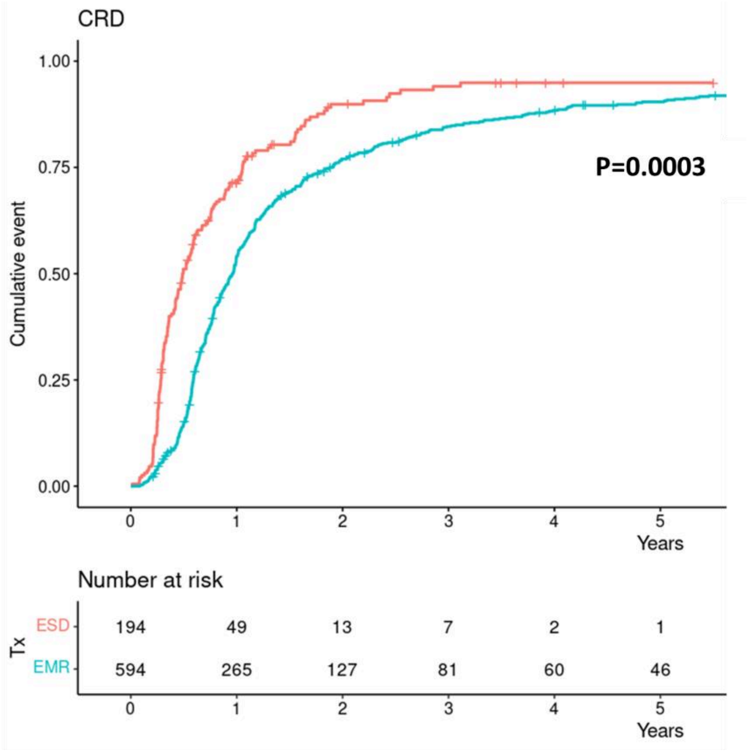

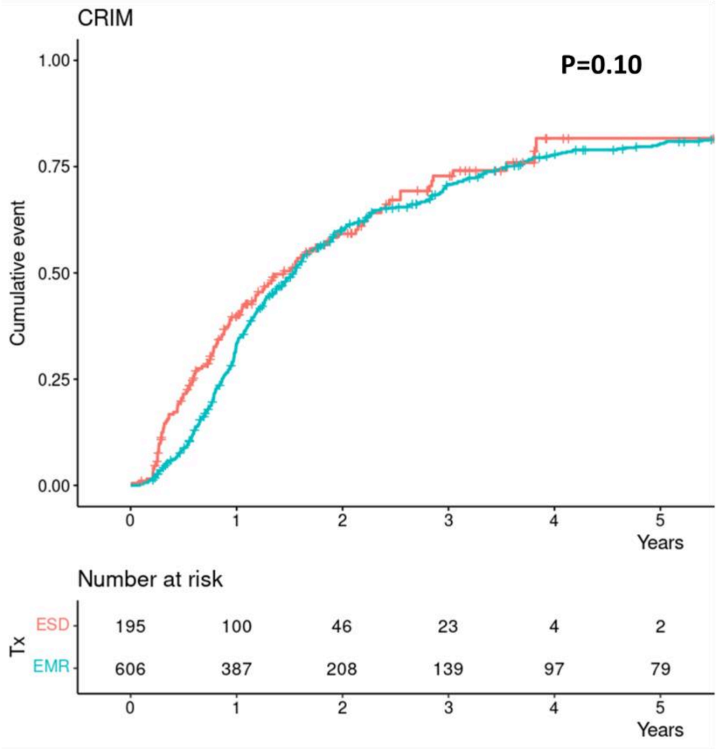

Supplement: Supplementary file 1 — Supplementary Material [file 10-1055-a-2422-2815_25542850.pdf]
